# Extra Supplement.—The Nursing Mirror

**Published:** 1894-02-10

**Authors:** 


### "The Hospital" Nursing Mirror.

Being the Extra Nursing Supplement of "The Hospital" Newspaper.

[Contributions for this Supplement should be addressed to the Editor, The Hospital, 428, Strand, London, W.C., and should have the word "Nursing" plainly written in left-hand top corner of the envelope.]

### News from the Nursing World.

### A ROYAL VISIT.

On Friday, February 2nd, His Royal Highness the Duke of York paid a visit to the West Norfolk Hospital at King's Lynn. He went through the wards and talked to many of the patients, in whom he showed a cordial interest. His Royal Highness expressed himself as particularly pleased with the children's ward which has been recently opened.

### THE ROYAL BRITISH NURSES' ASSOCIATION.

It will no doubt interest many readers to learn that in future representatives of the Press will be excluded from meetings of the Association and its Council. Our representative was given this information officially at the office of the Association a few days ago.

### CHELSEA HOSPITAL FOR WOMEN.

Dr. Louis C. Parkes has given a most alarming report of the condition of this hospital, and has recommended its being closed until its sanitary state has been amended. Scarlet fever amongst the nurses and a high rate of mortality amongst the patients are the outcome of the present evils.

### NO NEW THING.

The system by which the officials at rich St. Bartholomew's "reserve to themselves the power to levy a pretty heavy fine on any nurse who may fail to please them is not of recent date," wrote a correspondent last week, forwarding to us a printed copy of rules dated March 1st, 1877. The paragraph to which special exception was taken, under the heading, "Work Without Wages," in The Hospital of January 20th, stand in 1894 very much as they did in 1877, which proves the impossibility of their now being inadvertently inserted. Whoever first framed a regulation so cruelly unjust matters little; it is much more important that present and future nurses at this well-endowed hospital should be acquainted with the names of those who can courageously expurge for ever the obnoxious rule that "anyone failing to give satisfaction, either to the Matron or to the medical and surgical officers, will not be entitled to the payment; nor will any part of a current quarter's payment be made to anyone who, from any cause whatever, leaves the hospital in the course of a quarter."

### YOUNG WORKERS WANTED.

"A few hours a week, given regularly and punctually," that is the kind of help wanted from young workers, at the Children's Hospital and Home, 2, Maida Vale, where Miss Coleman is exceedingly glad to accept voluntary service, for the little ones nursed there are suffering from chronic or incurable diseases, and they are amongst the most helpless of all God's creatures. Many of them require everything to be done for them, and they are most skilfully and tenderly nursed in this home-like institution. On account of their exceeding helplessness, and also because of their long sojourn in hospital, they require amusement and instruction as well as good nursing. Surely there must be plenty of young ladies living at home who could devote a portion of their time to amuse and to wait on these poor little ones? Miss Coleman has a staff of voluntary workers, which often requires recruiting. Those willing to do this really useful work ought to take it up earnestly with the determination to let nothing interfere with their attendance at stated times. Intermittent help, which is often the amateur's idea of "being useful," not only disappoints but disarranges the regular workers, who have reckoned on steadfast helpers. Any earnest girl who wants to learn how invalid children are nursed, and to give kindly assistance in this incessant "care-taking," cannot do better than call and talk over the matter with Miss Coleman, 2, Maida Vale, W.

### MORE CHEAP POISONS.

A little boy died recently at Islington from the results of eating the contents of a box of sardines, price 2½d. It is no use to say that such food is "improper nourishment" for children, as little people and big ones, too, often crave for unwholesome, tasty condiments. The children of the poor have such monotonously untempting diets that it is natural that they should supplement their precarious home meals with whatever else they can secure. "Most for least money" chiefly attracts, and therefore it is the vendors who tempt children that require severe treatment. Still, nurses can prevent some disasters if they successfully impress upon their humbler patients the disastrous consequences of indulgence in cheap tinned foods.

### PRIVATE NURSING AS IT IS.

"Now, understand, nurse, I leave the patient in your hands; I've told the family that they are not to interfere. Her condition is most critical; you must not quit her for a moment." "Yes, doctor," replied the nurse, "I quite understand; I will do my best. How long must I watch her?" "Oh, all the time, of course. Good morning." "Please wait a moment, doctor. Is there to be a second nurse? I'm afraid after fourteen or sixteen hours I must get a good sleep myself, if I am to be of any use." The doctor looked puzzled. "Dear me! The people can't possibly afford another nurse, so you must do the best you can. There's an aunt, a sensible kind of woman, can take your place for a few hours to-morrow." Eighteen hours later "the sensible woman" came and relieved the nurse, remarking, "You won't want *all* the afternoon, I suppose?" And her smile faded at the quiet "I'm afraid I can't do with less than six hours' sleep." Eventually five hours were granted, and then followed twenty more hours of close work with the "serious case." "She does you credit," remarked the doctor on the fourth day. "Nurse's looks don't do *you* much credit," muttered the maid of all work, as she shut the front door. "I'm only a 'general,' but I gets my sleep and meals reg'lar."

### TWO COVENTRY INSTITUTIONS.

The Committee of Management of the Coventry Home Nursing Institution have issued their second annual report, which contains a satisfactory account of the year's work. It is found necessary to raise the fees charged for the nurses to thirty shillings a week, as the lower scale, of which trial has been made, proves insufficient to meet the expenditure. The district nurses at Coventry are getting on most satisfactorily, and much local interest is shown in both institutions.

### A LUCKY HIT.

"Please ma'am," said a cottager to a Health-lecturer the other day, "I've heard tell as you knows a deal about folkses insides, and I'd be main glad if so be you'd tell me of a cure for the 'digestion." "Well, Mrs. Brown, you know I never prescribe for anyone, but I should think you had better just be a little careful about what you eat and drink." "You've hit it, ma'am, you've hit it," responded Mrs. Brown with some excitement, "I know'd you would, and I've always fancied as food must have summat to do with 'digestion."

### QUEEN'S NURSES IN THE ISLE OF WIGHT.

The Ryde District Nurses' Association, which is affiliated with the Queen's Jubilee Institute, has furnished an excellent annual report of its seventh year of existence. Its work has been recently greatly extended, and the Inspector of Nursing was able to commend the management and method of the nurses, who are much appreciated in the district.

### COMFORTABLE ISOLATION.

The Sanatorium at Eastbourne is a home-like and pleasant place, devoid of the dreary characteristics which infectious hospitals are supposed to possess. A high wall shuts it off from the road leading to the glorious Downs, but an open gate seems to promise a hospitable reception. Through this gate, passing the porter's lodge, a gravel path leads to the front door of the pretty house where the nursing staff is lodged. It is a very comfortable and unpretentious building containing the official room of the visiting doctors, the matron's and nurses sitting-rooms and bed-rooms, a capital bath-room and a linen-room, the piles of snowy treasures in the latter testifying to the matron's excellent management and to a well-served laundry on the premises. A "discharge-room" is a rather recent and valuable addition. It contains the three customary divisions. The patient leaves all the clothes he has been wearing in No. 1, taking his disinfecting bath in No. 2, and habiting himself in fresh raiment in No. 3, from whence he passes at once through the garden into the outer world from which he has been so long zealously excluded. Diphtheria, typhoid, and scarlet fever are nursed in three separate blocks, the wards being light, bright, and orderly. Relations of the patients are allowed occasionally to inspect the latter through the numerous well-cleaned windows, thus obviating all risks of personal contact with infected persons. Improved bath-rooms for the patients and other additions are being discussed, and doubtless will be sanctioned, for it is evident that those in authority at Eastbourne grudge no pains to provide comfortably for all the reasonable requirements of the patients and staff.

### VERY HARD BOARDS.

The inmates of Builth Workhouse are certainly not to be envied. Seventeen or eighteen unfortunate tramps have to sleep on the bare floor without any coverings. The sick inmates have equally sad if somewhat varied experiences of the tender mercies of a Board. They are put in care of a woman who defends herself, not by denying the accusation of indulgence in strong drinks, but on the score that her unpleasant duties necessitate stimulants. She speaks of being on duty daily from five or six a.m. until ten or eleven p.m., and also going through the rooms at midnight. The appalling state of things thus revealed needs immediate reform. For a salary of £20 per annum this unlucky woman is expected to be on duty for seventeen to nineteen hours daily, the patients being apparently without any regular night nurse. A guardian is probably right in saying "if they gave the nurse notice they might have no applications for the office." But it is the duty of himself and fellow guardians to offer a salary which will attract a competent nurse to their sick wards, just as surely as they ought to give rugs and mats for the use of unfortunate tramps.

### HÔPITAL BOUCICAUT.

The district of Grenelle, which is one of the poorest and most thickly populated parts of Paris, is to have a fine new hospital of its own. The building will cover a considerably larger site than the one occupied by the Hotel Dieu, and will consist of eight distinct blocks. Sixteen rooms will be reserved for sick employés of the Bon Marché, the whole cost and endowment of the new hospital having been provided by Madame Boucicaut, the late generous and charitable owner of that huge establishment.

### INVALIDS ABROAD.

Influenza has kept nurses very busy at San Remo, and some of them have unfortunately been personally victims to this prevailing epidemic, a specially trying circumstance in a busy season of work amidst an invalid colony. Miss Bryant's Nurses' Institute seems to be making steady progress at San Remo, and her staff of trained nurses have been fully employed during the season. We hear of English nurses in every part of the world, but they should never go abroad without preliminary inquiries as to the prospect of success which awaits them.

### SHORT ITEMS.

Two scholarships of the value of £100 and £50 respectively are offered by a lady interested in mission work. The scholarships are tenable at the Edinburgh School of Medicine for Women, and are intended for women willing to educate themselves for medical missionary work.—Nurse Hopkins, of Longton Cottage Hospital, has been presented with a handsome gold watch by a number of friends in recognition of her valuable services and kindly attendance on the patients at the small-pox hospital.—It having been decided that the services of a trained nurse are much needed by the sick poor of Linlithgow, an influential committee has been appointed to consider the matter.—Nurse Mary Walsh, of the Nurses' Institute, Rusholme, was recently found drowned in the Rochdale Canal.

### On Nursing the Nervous and Insane.

By T. DUNCAN GREENLEES, M.B.Edin., Medical Superintendent, Grahams Town Asylum, South Africa.

### V.—HYPNOTISM.

HYPNOTISM, or the treatment of disease by suggestion, is one of the most modern forms of therapeutics, and in neurotic and hysterical females in France has proved of benefit in the treatment of such conditions. The Saxon is less impressionable to hypnotism than the Gallic type, and therefore the English manipulator has not the same mesmeric influence over his subject that his brother—the more effervescing Frenchman—has. This explains the fact that in the hands of English experimentors this treatment has not met with the same success that it has done apparently in France and even in Germany.

In speaking of hypnotism it must not be taken to mean that form of trance seen on the stage, when charlatans, for a monetary consideration, are prepared to go to sleep, and to do and say foolish things for the amusement of the audience.

Modern hypnotism consists in a peculiar state of mind in which the subject (or medium) is acutely alert to the suggestions of the operator, and the condition is generally induced by the temporary exhaustion of one or other of the senses—that of sight most frequently.

Without entering into the details of the process, which would take up too much time, it may here be stated that hypnotism has proved of great benefit in the treatment of many functional nerve disorders; local anæsthesia and hyperæsthesia occurring in hysterical subjects are amenable to its influence. Relief from pain, which at first is temporary, but, after repeated séances, becomes permanent when the cause is due to some functional disorder of the nerve, such as in ordinary neuralgia; and in a few cases of insanity the habits of the patients have been improved, delusions dispelled, the maniacal soothed, and sleep induced, all by suggestion, while the subject was under the hypnotic influence.

It might be almost imagined that in this treatment the cure had at last been found for neurotic conditions. Unfortunately, while admitting in certain suitable cases extraordinary results, only a very few persons are really susceptible to the influence of hypnotism, and it is only these very few who can participate in the advantages following this treatment. This explains the reason why, although its benefits are extensively published, and its cures noised abroad, many cases of neurotic disorders still remain to be treated, and so far the fame of hypnotism does not seem to be spreading.

The science (or "art" may it be called?) is still in its infancy when considered scientifically, although there is no doubt it was known to the ancients under different names; the influence of one mind over another has long been recognised, but so far as a thoroughly scientific explanation of the process is concerned, it is still in the hazy region of experimental research. A nurse is not likely to be asked to hypnotise a patient unless the gift is specially possessed by her. Then it is her duty to place herself entirely in the hands of the physician, and follow his advice most carefully with regard to using this influence over the patient, never forgetting that such a power is enormous in its influence for good or evil; therefore it must be carefully used and never abused.

### MENTAL DISEASES.

When speaking of the brain I mentioned the frontal region as the headquarters of the will, emotions, intellect, and passions; in other words, the frontal lobe is the seat of the mind. In a certain number of mental diseases no organic lesion in this portion of the brain can be discovered, and it is, therefore, compulsory to adopt the term "functional" again for this class of diseases. It is an extremely difficult thing to define the term "insanity," or mental disease. The most simple definition is that "it is a departure from the normal condition of one's mind," but this is not sufficient, and proves or explains nothing; what may be insanity in one person or race may not be insanity in another. Thus, foreigners have many curious customs, and the native races of South Africa have many strange ideas, which, if seen amongst English people, would be put down to eccentricity if not madness.

A person to be legally insane must be proved dangerous or troublesome to himself or others; if he is suicidal he requires to be protected against his desire to kill himself, and if he is dangerous to others society demands that he should be restrained from committing a crime.

Insanity is a generic term, and includes many different forms of mental disease; occasionally doctors speak of mania, melancholia, and so on, so that it is advisable for nurses to know something of the various forms to understand intelligently what is meant by these various terms.

When nursing a patient, however, it is most injudicious to speak of his illness or to give it a name. If the patient should not be too ill to understand, the thought of being insane is sufficient, in some cases, to interfere materially with recovery. To the ignorant public there is something repulsive and hopeless in the mere word, and the mention of insanity in some families is sufficient to stir up trouble.

Insanity has been classified times out of number; every writer has a classification of his own, and every classification presents some points to recommend it. That which I adopt is not original, but can be recommended from its simplicity:—

A. Forms of insanity depending on undeveloped brain.—(1) Idiocy, (2) Imbecility.

B. Forms of insanity depending on disease of the brain.—(1) Mania or mental exaltation—(*a*) Acute mania, (*b*) Chronic mania, (*c*) Delusional mania. (2) Melancholia or mental depression—(*a*) Acute melancholia, (*b*) Chronic melancholia, (*c*) Delusional melancholia. (3) Dementia or mental enfeeblement—(*a*) Acute dementia, (*b*) Chronic dementia, (*c*) Organic dementia. (4) General paralysis of the insane. (5) Insanity associated with epilepsy—(*a*) Epileptic mania, (*b*) Epileptic melancholia, (*c*) Epileptic dementia, (*d*) Epileptic imbecility.

It must not be supposed that this classification includes all the forms of mental disease; indeed, while we speak of "insanity associated with epilepsy" we might also mention the forms associated with every disease, both of brain and body, known; for all practical purposes, however, the above description will amply serve.

### Nursing in India.

### BENARES HOSPITAL.

THE nursing staff at the hospital at Benares consists of a matron and seven nurses. A scheme of training has been drawn up, and all accepted candidates are expected to remain at least three years at the hospital. If they work well during that time and pass their examinations satisfactorily, they receive certificates for general nursing and for midwifery.

### Minor Appointments.

CHERTSEY UNION ISOLATION HOSPITAL.—Miss C. Evans has been made head nurse of this hospital.

### Wants and Workers.

A HOME wanted for a young man without means, who is subject to severe epileptic fits. A small payment could be made.—E. C.

### District Nursing.

### VI.—DISTRICT HYGIENE.

### Pickles and Stewed Tea.

Many pennies are spent in pickles, small pieces of strong cheese, &c., which would buy much more wholesome food, and children are trained to require such additions to their meals. Salted fish, such as bloaters, haddocks, &c., are constantly purchased instead of the more nourishing and digestible fresh fish, like plaice, skate, &c. Endless farthings and halfpennies are wasted in bad sweets and cheap pastry, all bad for children's digestive organs. It is not so much the means that are wanted, but the knowledge to spend them on the proper food. Stewed tea is another fruitful source of evil both to adults and children. If after it is made it was poured off the leaves, it would be wholesome enough, but that is another point for the nurse to explain. If it could be realised that it was "medicine," not tea, when left too long, the mischief would be avoided.

One point the nurse can urge everywhere in town and country, viz., the necessity for boiling all milk and water before they are used for drinking. It is so simple, and yet saves so much; nine-tenths of the zymotic epidemics are conveyed in milk or water. It involves a little trouble and forethought, of course, but the result is worth it, and the reasons for the precaution must be forcibly brought home to the people.

Another fact constantly encountered by district nurses is that of children being suckled at the age of sixteen and eighteen months, and even up to two years old. This should be urgently represented to the mothers as most prejudicial both to themselves and their children. The custom has its source in a well-established tradition, and the mistake is very difficult to combat. But, like all these popular ideas founded on a small particle of fact, it is erroneous, and leads to incalculable mischief to mother and child. It is a source of gastric and intestinal troubles for the children, who are never as healthy as those weaned from ten to twelve months old. The drain upon the mother's constitution is too great, and weakens her permanently, thus reacting again unfavourably upon her children.

Many a rickety child might be saved its suffering, and the mother her care and anxiety, if she possessed the proper knowledge to bring it up healthily.

### Abuse of Stimulants.

The evil effects of stimulants on the delicate lining of a child's stomach can be graphically described, even apart from the moral evil of the acquired taste which will follow. Nothing is sadder than the frequent sight of little children of two or three being given tastes of father or mother's beer. The wickedness of dosing infants with gin to induce sleep is generally committed by mothers under the influence of "the drink" demon. This is the saddest and most hopeless class with which the district nurse comes in contact. So little can be done, for every maternal and womanly instinct is gradually effaced, and the pity of it! Insanitary dwellings and bad food have done more than anything else to swell the list of the victims to drink, the curse which saps the strength of the working classes.

When children are born and brought up in crowded airless rooms, with narrow, sunless streets to play in, fed on indigestible, unsuitable food, and taught to regard a "half-quartern" of brandy as the panacea for all complaints, and "a drop of beer" as the equivalent for a proper meal, what chance have they of resisting what in all probability is also an hereditary taste?

### The Best Weapons.

Cleaner, healthier homes, more wholesome, appetising food are weapons which every district nurse can bring to bear in the course of her daily work against the national foe. In posts where the pressure of actual nursing work is not too heavy, half an hour may often profitably be spent in practically demonstrating how a savoury meal may be prepared out of the most economical materials. The cookery learned at school should be encouraged at home, and the elder girls incited to prepare proper meals while the mother is laid aside. It benefits the household and trains the girls to apply their knowledge practically.

### Suitable Clothing.

With regard to clothing, the nurse can urge the necessity both for children and adults, but more especially for the former, of flannel or flannelette next the skin. In our variable climate chills are caught imperceptibly, and flannel vests, &c., minimise the risks.

Rickety children and those affected with diarrhœa should always wear a band of flannel round the abdomen, and this is useful for adults.

The custom of exposing infants and young children with bare necks and arms should also be waged war upon. Flannelette is very cheap, and little long-sleeved dresses or jackets worn under the low-necked dress can easily be made for 4d. or 6d. Most parents are alive to the necessity of good boots and warm outdoor wraps; but there is a curious carelessness in allowing children to run about for hours bare-footed and half clad in the house who would not be allowed to cross the street without a hat for fear of "catching cold." It is want of thought again, and the mothers are sorely troubled when an attack of bronchitis follows, and wonder "how it was caught." Another favourite custom is "to air" the family wardrobe between the flock bed and the mattress in sickness or health. If a mother understands these garments are soaking up the imperceptible perspiration and "holding the bad air," they will soon be removed and placed in their proper receptacle—the chest of drawers.

The practice of adults and children sleeping in the under-garments worn during the day is very unwholesome. Too frequently these are changed only once a week—not always then, and the effect is distinctly bad, especially for weakly children.

In her crusade against dirt and bad food, the nurse must "possess her soul in patience," and persevere in spite of the apparent hopelessness of the work. She is dealing with "children of a larger growth," and must be prepared to teach little by little, and as simply as possible. Every girl and woman roused to the importance of these details will be a centre for spreading them, and so nurses must be content to sow the seeds of better ways, and not give up in despair because no results are visible.

### The Nurses' Co-operation.

The annual meeting of the Nurses' Co-operation, 8, New Cavendish Street, was held at 20, Hanover Square, on February 2nd, and there was a large attendance on the occasion. The Hon. Sir Arthur Kekewich was in the chair, and excellent speeches were made by Mr. Thomas Bryant, Dr. Goodhart, Mr. Burdett, and others. Mr. Michelli read the report for the Hon. Secretary (Miss Morten), and a most satisfactory one it is. The Hon. Sir Arthur Kekewich gave an excellent address to the numerous nurses present, and specially urged on them the necessity for providing for a rainy day, expressing surprise and regret that they had not every one of them already joined the Royal National Pension Fund. He, in common with all the other speakers, testified to the admirable administration of the Lady Superintendent, to whom so much of the success of the Co-operation is due.

### Appointments.

Leavesden Asylum.—Miss Psyche C. Cronchley has been made Matron at this asylum. She has held the post of Chief Attendant at Whittingham Asylum, Preston.

### The Nursing of Ovariotomy Patients.

By J. Lawson Dick, M.B., C.M.Edin., Resident Surgical Officer, Clinical Hospital, Manchester, late Junior Assistant to Professor of Midwifery, Edinburgh.

### II.—THE NOURISHMENT.

With regard to the feeding of the patient great caution is called for on the part of the nurse. It is a good general rule that no nourishment should be given till flatus has been passed per rectum, and but little till the bowels have been moved. For the first twenty-four hours nothing is given. On recovering consciousness the thirst is great, but the patient must be encouraged to bear with it, as attempts to allay it simply increase the irritability of the stomach and aggravate the tendency to vomiting. It may be allayed to a certain extent by allowing the patient to suck the corner of a handkerchief dipped in cold water or to rinse out the mouth at intervals with cold water. Painting the hard, dry tongue with a solution consisting of equal parts of glycerine-borax and glycerine often proves very comforting. Great care must be taken by the nurse to see that her patient does not surreptitiously abstract ice from an ice bag should one be ordered, or drink the water from flower vases which may be near, so great in many cases is the desire for water. When twenty-four hours have passed and the patient shows no signs of sickness, a teaspoonful of warm water may be given at intervals of two or three hours. If this is well borne, and especially if flatus has been passed per rectum, at the end of thirty-six hours a teaspoonful of milk, beef tea, or chicken tea may be given every two hours. According to the patient's condition this is gradually increased. When the bowels have been moved on the fourth or fifth day a cup of weak tea with a small piece of dry toast will be much appreciated.

### Stimulants.

While this is the generally accepted mode of treatment, the nurse must be prepared frequently to break through it, and that without fear. Immediately after the operation, or during the course of the first day, if the temperature be very low with signs of collapse, or if it be high with quick irregular pulse, something must be given to tide the patient over the first twenty-four hours. If the call for stimulation be urgent, three or four hypodermic injections of brandy may be specially ordered. Ether should not be used. Better than this, however, is stimulation by means of the nutrient enema. Enemata consisting of one ounce of brandy or two drachms of Valentine's meat juice with ten minims of tincture of strophanthus may be ordered every two or three hours if necessary till the danger has passed. If necessary brandy or champagne may have to be given by the mouth in teaspoonful doses. No good purpose is obtained by pouring in large quantities of stimulants. After the second day the patient may be washed all over, hot-water bottles being kept about her during the process. On the second and third day flatulent distension of the abdomen is apt to be very troublesome. This is best avoided by seeing that the alimentary canal is in good working order before operation by giving nothing for the first twenty-four hours, and by avoiding the use of morphia. If the abdominal distension is great, the temperature should be raised and not brought down on applying an ice-bag, and especially if after the passage of the rectal tube no flatus is passed, it will be well even as early as the evening of the third or fourth day to move the bowels. This is best done by means of Henry's saline aperient, consisting of half an ounce of sulphate of magnesia dissolved in one drachm of dilute sulphuric acid and one ounce of water. A small enema containing ten grains of quinine is sometimes very effective in inducing the passage of flatus. In every case the bowels should be moved by the fifth or sixth day. The tendency is always to delay this too long. Here, again, Henry's solution is the best aperient. After the bowels have been well moved, solid food may be cautiously given beginning with the addition of a little tea and a small piece of dry toast, and passing on to milk pudding for dinner, later to chicken, and so back to ordinary light diet. The bowels should be regularly moved every second day. Glycerine injections will often be useful. Usually there is slight vaginal discharge appearing about the third, fourth, or fifth day. This may be soaked up by means of pads of wool, and the parts should be cleansed twice or thrice daily.

### Troublesome Cases.

Very troublesome cases are those occurring in elderly or debilitated women when the circulation is feeble, or when aortic or initial disease cause backward pressure and congestion of the pulmonary circulation. Here the temperature on the third or fourth day rises to 101 deg. or 102 deg. F. There is troublesome cough, with difficult expectoration, and the patient looks in a very precarious condition. The condition is one of hypostatic pneumonia, the basis of the lungs posteriorly being the seat of a low type of inflammatory process. The onward flow of the blood is interfered with, and it consequently tends to gravitate towards these parts as the patient lies on her back. The first and all-important indication then is to vary the position of the patient. As a rule, bolstering her up with pillows till she is in more or less of a sitting posture is very effective, as also is allowing the patient to lie on one side or other after she has been carefully helped round by the nurse. Brandy by the mouth and by the rectum may be ordered, also five minims of the tincture of digitalis may be prescribed every four hours with advantage. As much as half an ounce of brandy or good whiskey may be ordered to be given by the mouth every two hours after the fourth or fifth day. About the ninth or tenth day stitches are taken out, but frequently the surgeon looks at the wound earlier. When the temperature keeps above normal but the pulse is regular and slow, the great probability is that a stitch abscess is forming. In such a case the wound will be dressed and the offending stitch removed. After the stitches have been removed the wound should be dressed daily until it is perfectly dry and healed. The abdomen should then be securely strapped by bands of sticking-plaster. After the first week is over, if the patient's condition is satisfactory, the night nurse may be dispensed with, and if in hospital the patient may be moved back into the general ward. During the second week the patient should be measured for an abdominal belt. At the end of three weeks she may get out of bed for a few minutes. Not much should be attempted the first day. If she stands this well, on the following day she may have a bath.

### Cautioning Patients.

When in hospital the patient is, as a rule, dismissed at the end of the fourth week. Before she leaves medical guidance detailed and explicit rules should be laid down for her to follow. When five or six weeks have elapsed the sticking-plaster may be dispensed with altogether, and the only dressing required is a small pad of wool between the belt and the wound. The belt is, of course, removed when the patient is in bed. The patient should be strongly cautioned that for at least twelve months she must consider herself more or less an invalid. Much of the neuralgic pain and general ill-health so frequently erroneously put down to the fault of the surgeon is in reality due to the want of attention to instructions on the part of the patient herself. After twelve or eighteen months have elapsed the patient may, if she choose, dispense with the abdominal belt, the scar by that time being sufficiently consolidated to obviate any tendency to stretching of the cicatrix and hernia'ion of the bowel.

### Nursing in the United States.

### OUR NEW YORK LETTER.

### The Superintendents' Convention.

Our Superintendents' Convention went off very well, and we organised into a society to meet once a year. Forty-five superintendents of representative training schools attached to general hospitals assembled. They came from Chicago, Cincinnati, Indianapolis, Baltimore, Maryland, Philadelphia, Boston, Providence, New Haven, Toronto, Ottawa, Montreal, Brooklyn, Jersey City, and other smaller cities, including Buffalo and Rochester. Those who understand anything of the geography of our country will comprehend that many thousand miles were covered to bring these superintendents together, and will appreciate the *esprit de corps* thus manifested among members in the profession.

We did little but organise at this first meeting, agree on a constitution, and get acquainted. The social features were not by any means the least important part of the convention. The managers of the Bellevue Training School gave a reception to welcome the superintendents; several of the visiting physicians connected with the large hospitals in the city also gave receptions. A luncheon as well as a dinner took place in one of the "swell" hotels. These were given by the superintendents of Brooklyn and New York to all the superintendents; and several small dinners were given to groups of a dozen or so superintendents.

Our guests went back to their respective training schools well pleased with the convention in all its aspects. The Organising Committee, appointed at the Chicago Congress, were Miss A. L. Alston, Superintendent Mount Sinai Training School, New York (President); Miss L. Darche, New York City Training School, B.I., New York (Secretary); Miss L. L. Drown, Boston City Training School (Treasurer); Miss Hampton, Johns Hopkins Hospital, Baltimore; Miss M. Davis, University Hospital, Philadelphia; Miss Palmer, Garfield Memorial Hospital, Washington, D.C.; and Miss Sutliffe, New York Hospital Training School, New York.

### BETHLEHEM, PENNSYLVANIA.

### By Our Own Correspondent.

Bethlehem is a picturesque town situated among the hills, full of active life and enterprise, and has much of historical interest to commend it to notice. It is such a large railroad centre that St. Luke's Hospital, standing on an eminence at South Bethlehem, is a great boon to the employés on the line.

St. Luke's Hospital is free to all, without regard to colour, nationality, or religious creed. The charter of incorporation was granted this hospital in 1872, and in the year following it received its first patient. The present structure, erected in 1876, is in the Pavilion style, and the grounds extend over forty acres. The entire staff of workers is lodged in the administration building.

The hospital has been for twelve years under the control of a single responsible man, who is not only the general executive officer, but chief surgeon and physician. Associated with him are two assistant resident physicians. The system of placing one person in charge of all the workings of the hospital was established, and has been worked out by business men with most satisfactory results.

During the past ten years 3,757 patients have been treated in the hospital, and 10,794 in the dispensary department. The total number of days of treatment in hospital for ten years was 96,632, and the total number of visits paid to the dispensary was 37,173. Combining the total number of days in the hospital and the total number of visits to the dispensary would represent the equivalent number of individuals the hospital has treated for one day during ten years, namely, 133,805. This has cost the hospital 157,854.50 dols. This includes all the expense of the institution except building operations and interest on indebtedness.

Since 1885 this hospital has maintained a training school for nurses, and the expense of this department falls in the main upon the general account of the hospital, and consequently the expenditure above-mentioned includes that of the school. The average daily cost for running the entire establishment for ten years has been 42 dols. and 95 cents (42.95 dols.) Taking the sum of the whole number of days in the hospital and the whole number of visits to the dispensary as a proper co-efficient, would give a daily per capita cost of 1.17-100 dols. Or, computing it only for the patients actually treated in the wards, would give an average per capita cost of 1.62 dols. a day.

Not including the cases admitted in hopeless conditions, the average mortality has been 4·41 per cent. There have been 1,370 surgical operations performed, with an average mortality rate, after operation, of 2·54 per cent.

The standard for entering the training school is very high, and the number of applicants far more than can be accepted, as the number of pupil-nurses is limited to 14. The terms of the hospital year, so far as concerns the training school, begin on April 1st and October 1st, and the course of instruction embraces two years.

The Principal of the training school exercises the functions of her office subject to the general authority of the Superintendent. With this reservation the school is under her direct supervision and control, and her authority extends over all that pertains to the duties and discipline of the nurses in the wards as well as to the details of their instruction in the school. All the servants and orderlies are responsible to her in the performance of such duties as relate to the nursing of the patients.

A monthly allowance of eight dollars for the first twelve months is made to each pupil, and fifteen dollars during the remainder of the term. The instruction is given by the medical officers of the hospital, by the principal of the school, and by the head nurse of the ward, each ward being provided, so far as possible, with a head nurse, senior assistant, and junior assistant. During the first year of instruction and practice the nurse is a pupil and on duty as assistant nurse in the hospital, and in the second year she is on duty as nurse either in the hospital or in private families.

### Presentations.

An interesting ceremony took place at the Lambeth Infirmary on Monday night, when a testimonial was presented to Dr. Blachford, assistant medical officer, on the occasion of his departure for the City and Borough Asylum, Bristol. Among the company were Mr. W. Stockbridge, L.C.C. and Guardian, Dr. Smith, Mr. Martin (steward) and wife, the Rev. T. H. Leale, the Rev. Father H. Cafferata, Miss Griffiths (Matron), Mr. G. Dean (assistant steward), Mr. Blachford, sen., Mr. B. Austin, Mr. S. Austin, Mr. H. Eilbech, Miss Hicks, Miss May, the nurses, and other officers. A handsome clock, with bronze ornaments and an inscription on a silver plate, and a travelling bag were presented to Dr. Blachford, and the Chaplain, the Rev. T. H. Leale, and other gentlemen made appropriate speeches. Dr. Blachford takes with him the cordial good wishes of Guardians, patients, and officers.

On Monday evening, January 22nd, the committee, medical staff, and friends of the Eston Hospital met to present Miss Rothwell, the late matron, with a handsome afternoon tea service, salver, and a sum of money. Mr. John Anderson presided. Mr. John Thomson, vice-chairman Eston Hospital, made the presentation. He stated that during the nine years Miss Rothwell had been matron she had, by her tact, capability, and assiduity, endeared herself to one and all of them, as the very handsome testimonial before them proued. He trusted that she would be long spared to do useful work on a large scale in her new sphere as Matron to Rochdale Infirmary, and that the testimonial would make her think of the many friends she leaves at Eston. Dr. John Gem spoke on behalf of the medical staff, Mr. Webb on behalf of the committee, and the Rev. E. T. S. Besley, vicar, and the Rev. Father Nolan on behalf of the visitors and friends to the hospital. Miss Rothwell leaves Eston with every good wish for her future success in the profession she has chosen and for which she is so suited.

### Everybody's Opinion.

[Correspondence on all subjects is invited, but we cannot in any way be responsible for the opinions expressed by our correspondents. No communications can be entertained if the name and address of the correspondent is not given, or unless one side of the paper only be written on.]

### NURSING SMALL-POX.

"ANOTHER NURSE" writes: I think "A Nurse" will find that the most reassuring reports of medical officers will fail to dispel the natural horror which folks have of nursing small-pox. I believe that only practical experience will ever prove to them how safe it is for those properly protected by re-vaccination to nurse this loathsome disease. I remember being very nervous on going to my first case, and after screwing up my courage to oblige my matron, I felt it a great grievance to find the patient convalescent. I was rewarded, however, for my courage in a very few days by having several patients down with it. When I had been nursing them for some time I told the doctor I had never before seen a case; he appeared much surprised, but informed me that neither had he!

### NURSING OVARIOTOMY PATIENTS.

A "SENIOR NURSE" writes: Mr. Dick's novel ideas upon the nursing of the above cases certainly invite some remarks from senior nurses; those, for instance, who for the last twenty years have successfully worked under distinguished surgeons. I have always seen the surgeons assist in putting the patient into bed, but not in the sheet, which generally is damp, to say the least of it, when the operation is concluded, and no one could be ignorant enough to try to put a fresh one under the patient. Where is the one hand to be put over the abdomen when the patient is sick? One on either side of the wound was the order always given to me. Does a surgeon ever put on a bandage that can move, and would he permit any nurse to touch a dressing for the first twenty-four hours in these antiseptic days? The giving of morphia rests with the surgeon and not with the nurse. A patient who has a quarter-grain hypodermic as soon as she becomes restless generally sleeps off the shock, and healing by first intention also commences. The idea of suggesting to a patient that she should exert herself to pass urine naturally may, of course, be done, but surely no nurse would venture to pass a slipper under a case for forty-eight hours, for what would the consequences be if a glass tube was in the wound?

### MIDWIVES' REGISTRATION.

DR. F. H. ALDERSON writes: I received a letter this morning from a medical friend who has given much attention to the question of the need and the wisdom of legislating for the control of the practice of midwives, requesting me to reply to the strong partisan letter of Dr. Hugh Woods, whose letter is evidently written under annoyance or irritability, for he, Dr. Hugh Woods, "Master of Midwifery," has said, "there is no need for a Midwives' Bill, and there shall be none." I, fearing to fall in a like error of writing on the spur of the moment, and doubting if it required a reply, laid his letter aside, believing my views, which are unchanged on this somewhat heated question, are well known. It is somewhat singular if the whole of the members of the Medical Practitioners' Association think with Dr. Hugh Woods and not at all with me that I should have been unanimously elected their president, and especially as I expressly told the Council when the honour was proffered me that I advocated wise legislation for the control and supervision of the practice of midwives and that I was a member of the Midwives' Registration Association, but I certainly added, that I advocated they should be registered as obstetric nurses, and this I still maintain, and refused to sign the letter issued by the council of the Midwives' Registered Association containing the paragraph that the present *bonâ fide* midwife should be allowed to continue her calling, hoping, as I do still, that the designation midwife may ultimately be dropped altogether. He is a bad politician who will refuse a useful measure of reform because it is not exactly as he would desire it, and I am afraid that Government would not think the vested right of the present midwife sufficiently cared for, if only allowed to call herself an obstetric nurse, she can do less harm by being placed on a separate list as proposed by the Council of the Midwives' Registration Association, than totally irresponsible as she now is. When a candidate for the honour of a seat as a direct medical representative on the Medical Council, I said in my published address, "Midwives should only be allowed to act when a doctor had been already engaged, and thus be under the eye and responsibility of the medical man, and if the midwives are registered, they should be not as midwives, but as obstetric nurses, and this I advocate as strongly as ever, and have repeated in page 3 of my pamphlet on "The Danger of the Non Regulation of Midwives." Indeed, I spoke as to the need, in the interest of the public, of legislation at the meetings convened by the British Medical Association at the School of Arts in Jermyn Street, last autumn, but such legislation must not be, and need not be, inimical to the interests of the great profession to which it is my honour and pleasure to belong. If my opinions and views even on the question of the legislation for midwives were opposed, as Dr. Hugh Woods infers, to the great majority of general practitioners, I should hardly have received with very little effort on my own part 4,816 votes at the last election of direct representatives of the Medical Council, but I think Dr. Woods has spoken too much for himself; at least, I know of others who think as I do, and who are quite certain of the earnestness of my efforts to advance the interests of the general practitioner, or I would resign the office, great as I esteem the honour of being president of so important a body as the Medical Practitioners' Association; but I am encouraged to believe I have the approval of the profession, notwithstanding the letter of Dr. Hugh Woods, for the secretary writes to me: "We are increasing very rapidly in numbers; we have had some 50 new members the last ten days or so?"

### Novelties and New Preparations.

### DRESS PRESERVERS.

(CANFIELD RUBBER COMPANY, 64, BASINGHALL STREET, E.C.)

WE have on previous occasions drawn attention to the excellent Canfield specialities. The dress preservers now before us have been so long and deservedly popular that it is not possible to say anything new on the subject. We have not met their equal so far, and were it recognised that through their durability they are in effect the cheapest in the end, there is little doubt that they would supersede all others. They wash excellently, are soft and warm, and though waterproof they lack the disagreeable odour usually associated with indiarubber goods.

### A NOVEL PREPARATION OF CHEESE.

(THE GLOUCESTER DAIRY ASSOCIATION.)

The Gloucester Dairy has made a distinctly new departure in the Savory and Anchovy Cheese which it is now producing. These condiments take the form of potted cheese, variously seasoned as their names suggest. Cheese does not bear a high character for its digestive qualities, but those who have a partiality for it are inclined to risk ill effects. In this new form a very little goes a long way, and it is a most savoury and dainty addition to luncheon or supper fare, and we fancy that the public will speedily take it into favour when once it is introduced amongst them.

### LIMONA.

(MESSRS. L. RAWSTHORN, GREENBANK MILL, PRESTON.)

Limona ranks with the foods now so much in vogue, which are carefully prepared with a view to combining all the ingredients requisite to form a nutritive diet with an agreeable taste in a digestive form. It is composed of whole grain and is rich in hypophosphites. From it excellent puddings, porridge, gruel, and biscuits can be made at a small cost, and it will be found to be a decided addition to the store-room.

### Women's Work.

### THE RESIDENT GOVERNESS.

We are hearing so much at the present moment of daughters who want "to do something" and to have the same liberty as their brothers, that a glance at some of the spheres of work that are open to the young woman of the present day, and for which she supposes her soul to be yearning, may not be out of place.

We will suppose our young woman to be a lady by birth, and in all ways thoroughly refined, and with excellent health; to have had as good an education as it is possible for a girl to have (and this means a good deal, it means having obtained certificates for various examinations, and having had the benefit of the best masters for what are usually termed accomplishments). She is consequently well equipped, both mentally and bodily, to fight life's battle.

Now what sort of a life does she find ready to her hand? A home, possibly, where the means are sufficient and where everything is well ordered and comfortable, but where there is probably not much margin for such luxuries as travelling, theatres, balls, &c.

This home consists of two sisters, besides the father and mother; the mother still young, with plenty of energy for her housekeeping, and with years of experience, which make her energy go double the distance.

The daughters yearn to be useful, and find there is nothing for them to do, and, with the best intentions, they do but tread on each other's toes. There seems no room for them and their big ideas at home, and every outside occupation which they might take up, such as attending lectures, &c., means spending money; and their education being nominally finished, there is possibly a difficulty about having the money given them. What else can they do? Afternoon teas will not fill the gap, and they have too much principle to spend their time on the sofa with a novel.

Then comes the question, what are they to do? for to do something with the talents that have been given them, and which have been so highly cultivated, seems an imperative necessity. Teaching perhaps appears as the fittest career to them.

We will glide over preliminaries, and will suppose one of the girls established in a family, "titled," perhaps, and who pay her well, *i.e.*, £80 or £100 a year; so far her longings are fulfilled, she has become a worker.

And now come the stern realities; her mind, which has been fed upon advanced subjects, has to come down to elementary ones, and these she has to keep on teaching day after day; patience being of more practical benefit to her than knowledge.

Her intercourse with her grown-up fellows consists of the twenty minutes or so during which luncheon lasts, and this same intercourse rarely rises above a few casual remarks of "The storm seems to have done a good deal of damage" order.

When visitors are present even this is not to be had. She may, or she may not, be introduced to the person sitting next her, and at most she has to answer a kindly condescending remark.

To a woman brought up to anticipate the life of a governess no doubt this may not appear irksome, but to one who takes to it as "work" this kind of remark is gall and wormwood, coming as it does from women or men in no way better bred than herself.

This one break in the day being over, she falls back upon the undeveloped minds of her pupils, and as time goes on she begins to feel as dull and cramped as her surroundings, or rather of that part of them which is all she has to do with.

The children themselves may be quite average specimens, tolerably good and obedient, until the whim seizes them to be naughty; they may be "talked to" or punished, as the case requires, but any correction usually results in their banding together to commiserate the culprit and to put the governess in Coventry. None of them speak to her beyond saying yes or no during meals and playtime until a day or two has gone by, when the usual state of things is resumed, until the next occasion comes for punishment, when the experience of Coventry is again repeated.

And these children are good specimens, with no actual vice about them.

Home teaching differs so much from school training. At school the common opinion of the majority forces a girl to behave politely to her teacher, and to do her work properly; but at home there is no incentive to work or to be polite if the pupil does not feel inclined. She knows she can be punished, but she also knows that her parents will consider that "Poor Miss X. cannot manage the children, as she seems so often to have to punish them."

The people may be kind enough (excepting such types as the "mamma" in Mr. Anstey's charming "Young Man from Blankley's"), but the governess to them is seldom anything more than a paid outsider. Like Thackeray's governess, she even does her worshipping at morning prayers "a little apart," and it is this perpetual apartness that seems in time to eat into her soul. There is no comradeship, no intellectual height to strive after—all that is required of her is to be patient and to endure.

The strain seems to take from her all inclination to read anything beyond the newspaper during the one or two evening hours that she has to herself.

She dines in solemn and solitary state off several courses; women as a rule do not care much about their food; and, oh! how often would she like to exchange this "stalled ox" dinner for one of herbs seasoned with a little love. But she has chosen her lot, and like the old hero, Sir Richard Grenville, she "sets her face and fights on."

Years of this kind of life may go on; she gets to be more tolerated by her pupils, who in time possibly come to consider her in the light of a friend—ever patient and ever ready to help and to listen to them—but this does not come until she is on the verge of leaving them, and when her pupils are emerging into the great world; and then, and this is the rub, the whole performance has to be gone through all over again.

A finishing governess remains at the most three to four years in one family, and then she finds her work accomplished, her pupil is emancipated, and she has to look out for pastures new.

How much better off is the cook or housemaid? Meals always want cooking, and beds always want making; at no period can they ever be said to be "finished."

Yet this kind of household would be considered as an eminently desirable one; the supposed governess worked with the intention of making the best of everything and of doing her duty. Now let us consider results.

Her bodily health has not suffered, it has possibly improved; but she is conscious of possessing "nerves," and her mind which she sought to employ, if not to improve, seems to have gone out to grass. She has saved a little money, perhaps, and she finally, after several years of work in perhaps half a dozen different families, comes home to find that her place knows her no more (her short holidays having seemed always merely like visits). She has given the best years of her life to strangers, and she now returns to find love and quietness certainly, but also a life whose interests and thoughts she seems entirely out of touch with.

She has become orderly and precise, decided and perhaps somewhat "masterful"—it was her trade, and she was required to be a disciplinarian; but now she finds folk somewhat shy and afraid of her as a friend; the very qualities that made her such an excellent governess, and which she acquired with such pains, seem now to stand in her way.

Fate appears a little rough on her, and her life cannot be said to have been either very happy or very pleasant; and yet it was what she wanted, and what she chose to do, perhaps in the face of opposition if not of disapproval.

E. G.

☞ For The Muses' Looking Glass, Book Reviews, A Sad Occurrence, and Reading to the Sick, see page clxxxix, et seq.

### The Muses' Looking Glass.

### WOMEN WRITERS AND THEIR WORKS.

MISS CATHARINE HAMILTON'S "First Series of Women Writers"* was so favourably received by the public that she "was encouraged," she remarks in the preface of the present volume, to offer a "second" series, in the hope that the public would find it of equal interest as its predecessor. And that the writer has fully succeeded in her object will be readily admitted by all, and she carries on in an admirable manner the tradition so happily begun in her first work. If, amongst the women writers with whom Miss Hamilton deals, few of them, with one or two notable exceptions, could afford to have their writings judged by the highest literary standard, that is not a fault to be laid against their compiler. Charming, indeed, as is much of their work, it would barely stand the crucial test of an unprejudiced criticism. The fashion of to-day denies a place on the parlour table to the tearful plaintive verse of the songstresses of our grandmother's time. The serious, sad-minded tone of women writers of the first half of the nineteenth century has given place to one more morbid, personal, and introspective; whether the writings of these earlier verse-makers will survive, and be known to succeeding generations save in name, is a question which posterity alone can decide.

First among these chatty biographical sketches, which Miss Hamilton arranges in chronological order, comes that of Mrs. Hemans, the favourite poetess of her day. Her popularity, dating early in the commencement of the present century, has not gained with time. A mother's love, the fidelity of a child, the beauty of spring, the sadness of early death, and the hope of heaven are the most congenial subjects of her pen, but if Felicia Hemans was neither a Sapho, nor a teacher of men, still she had a certain sweet way of her own—a manner of expressing herself which has found no rival since; there is, indeed, a ring of true poetry in her productions to which "thin vein," as he calls it, Carlyle refers in criticising her verses, when the writer was but a youthful aspirant to fame. Mrs. Hemans had an interesting circle of literary friends, amongst whom were Mary Howett, Sir Walter Scott, and William Wordsworth. The latter it is who pays the poetess a high tribute of respect when, at her death in 1835, he laments her as "a holy spirit:—

> Sweet as the spring, as ocean deep,
> Who ere the summer faded
> Has sunk into a breathless sleep.

Another instance of the many evidences which are furnished us throughout the wide range of literature and art, of how fleeting is the wave of popularity, is exemplified in the case of Fredericka Bremer, who first saw light in the chilly climate of Finland. The fire of public applause, the burst of enthusiasm with which the works of this biographer of the joys and sorrows of Northern life was met with, at the time of their publication, burnt and wore itself out at a proportionately accelerated speed. Few, alas, amongst us now know the good Fredericka except in name.

The same, too, is seen with Letitia Elizabeth Landon. She also was welcomed with a burst of praise; by contemporary critics she was even compared to Shakespeare; her poems, which were signed with her initials, L. E. L., by which she is still known, had a circulation only equal to "Lalla Rookh." Her prose, too, later on, showed immense power of wit and satire, full too of quaint humorous observations on the times in which the writer lived. One novel which appeared in 1831, entitled "Romance and Reality," is an abundant evidence of the powers with which the gifted authoress was endowed. Now a days, and more is the pity, there is small demand for the quaint, shrewd tales of Letitia Landon.

A happy exception to the short-lived fame of popular writers of their day is Elizabeth Barrett Browning. As Miss Hamilton says, "We are continually meeting scraps of her poems in sermons, in essays, in speeches, whenever men wish to stir the hearts of men. Those who learn from her cannot but feel ennobled and elated by so doing. The 'touch of Christ's hand' was upon her. For her life, for her teaching, for her music, for herself, we can never be sufficiently grateful." The municipality of Florence placed a white marble slab on the wall of Casa Guidi, when this poetess died, the words of which run as follows:—

> Here wrote and died
> Elizabeth Barrett Browning,
> Who in her woman's heart united,
> The wisdom of the sage and the eloquence of the poet,
> With her golden verse linking Italy to England.
> Grateful Florence placed this memorial A.D. 1861.

The middle of the 19th century saw two women busily writing, one in a gloomy Yorkshire parsonage, the other in the crowded city of Manchester. The one was Charlotte Brontë, the other was Mrs. Gaskell. Their lives, their associations, were totally different. The rush of life in that busy city where she lived is told through the writings of Mrs. Gaskell. She made common cause with Manchester operatives and factory hands; a sympathy, which besides assuming practical proportions, extends through the medium of her pen. Of another order, and one less subjective, are the writings of Charlotte Brontë, more passionately written are these, with a burning, eager intensity, and by reason of their strong personality and original vigour have long outlived their author. "Jane Eyre" and "The Professor" have found no rivals in succeeding literature. Highly imaginative, strong, Charlotte Brontë's novels move us in a mysterious way individual alone to themselves. Under the soubriquet of "Currer Bell" it is Charlotte Brontë who talks; through the print her spirit seems to hold converse with us. It was through the medium of this expression that the woman gave her soul its natural release—through her writings. The difficulties under which she laboured have onlyadded laurels to the wreath which posterity laid on the grave of Currer Bell in that desolate country churchyard.

Miss Hamilton could not have found George Eliot's life an easy task to condense into a few short pages. It is an especially difficult career to follow, even when exhaustively treated; in a limited space the difficulty necessarily would be increased. "In her life, as well as in herself," as her present biographer remarks, "we find many startling contradictions, many unexplained inconsistencies. She was at once diffident and ambitious, nervous, easily depressed . . . and yet with a keen, clear vigour of intellect more masculine than feminine." To get any real glimpses of the inner life of this many-sided genius, whose fame is immortalised under the *nom de plume* of George Eliot, we must go to her journals and letters, which were edited by her husband, Mr. Cross; her novels have but little of the purely "personal" note about them.

When we close Miss Hamilton's volume, the thought forces itself upon us, how hard it is, and how rare it is, to judge impartially of women's work. For to estimate their work as woman's work is at once to restrict the domain and limit the empire. To judge from a standpoint wherein sex is no motor, to separate the work from the worker, that alone is general criticism. Currer Bell and George Eliot, and since their date many others, have shown to the world the great things which female penmanship are capable of.

Art is neither a question of sex or of nationality; and in all criticism it must be judged apart from such conditions, whilst in so doing old prejudices must give way and false premises be dismissed.

* "Women Writers: Their Works and Ways." Second Series. (London: Ward, Lock, and Bowden, 1893.)

### The Book World for Women and Nurses.

[We invite Correspondence, Criticism, Enquiries, and Notes on Books likely to interest Women and Nurses. Address, Editor, THE HOSPITAL (Nurses' Book World), 428, Strand, W.C.]

BIBLEWOMEN AND NURSES' MISSION.

THE Mission employs 130 Biblewomen and 82 nurses in all parts of the metropolis, under the guidance of 150 lady superintendents; 41 Biblewomen and two nurses are affiliated, and engaged in similar work abroad. This is a record of many years' quiet unobtrusive, persevering work, not the less remarkable because those who are most concerned in it do not strive, nor cry, nor cause their voice to be lifted up in the many streets in which they so patiently labour. The work of the Biblewomen is rather difficult to define, for its success is so largely dependent upon personal influence. She has five great objects in view: (1) To supply Bibles in poor districts to all who can afford a small weekly payment; (2) to gain a personal influence over those visited, to read and pray with them when possible; (3) to invite poor women to a mothers' meeting; (4) to visit the sick and dying; and (5) to win the mother and the home for Christ. Many touching instances are given in the record, showing how the Biblewomen are used by the great Master, not only to help and comfort their poor sisters, but in building up of Christian character in many a poor home. The only question we feel inclined to ask is whether the Biblewomen receive any previous training before venturing upon their spiritual work corresponding to the systematic education received by the nurses. The work of dealing with souls is no less complicated, difficult, and disappointing than that of ministering to the body; nay, is it not more difficult? If so, the need of a definite training through competent teachers is great indeed; however loving a Biblewoman may be, however fully she may be persuaded that she is called to the work, impulse, unless supplemented by long and careful training, will not be sufficient.

In the nursing branch we notice that the utmost care is taken (1) to provide trained nurses, who attend the sick poor in their own homes; (2) to maintain a sufficient staff of district superintendents, who visit with the nurses and report difficult cases at headquarters; (3) to supply nurses with medical stores and food to dispense; (4) to maintain at Southend a seaside home for convalescents. The key to the success reported in this branch is, of course, the same as in the other, viz., personal attendance at the home. The poor feel this very deeply. Even if they procure a large measure of medical skill and a series of comforts at a hospital which they cannot have otherwise, there is a something about being nursed "at home" by a sympathetic person which surpasses any other treatment, and which meets with gratitude and appreciation known only to those who have the privilege to minister in this way to the Body of Christ. "I was sick and ye visited me."

SURGICAL WARD WORK AND NURSING. By ALEXANDER MILES, M.D.C.M., F.R.C.S.Edin. (The Scientific Press, London.)

"Surgical Ward Work and Nursing." The author of this admirable little work has ably appreciated the needs of nurses, and he has given them a *vade mecum* of invaluable interest and assistance; he has greatly enhanced its value by the practical and simple manner in which he has treated matters of interest and importance to those working in the wards of our hospitals, giving information not generally found in the many excellent text-books that have been written for the use and guidance of nurses.

The laws of surgical work and the principles of Listerianism "predominating over all other methods of wound treatment" being clearly set forth, will at once realise to the appreciative nurse the importance that is attached to the necessity of care on her part.

The general principles of antiseptics; the advantages and disadvantages of various lotions, their preparation, powders, ungents, and dressings in use are clearly explained; Anæsthetics, their physiological action, method of administration, dangers and their treatment; the use of rest in surgery, and many other matters of great value to nurses where their responsible duties are concerned, are broadly placed before the reader.

Dr. Miles is to be congratulated in so successfully meeting a long-felt want.

---

MAGAZINES OF THE MONTH.

THE ENGLISH ILLUSTRATED MAGAZINE for February is what may be called a good "all-round" number. Mr. Laird Clowes discourses on the British Navy, and Mr. Dudley Heath on "Coster-land." "A Queen as Mountaineer" is a description of the summer retreat of Queen Margherita of Italy, Gressoney St. Jean, in the valleys of Piedmont, and an account of the mountaineering exploits of her active Majesty; and there is much that is interesting in Mr. George Moore's lively and not altogether flattering paper upon Zola. According to Mr. Moore, the author of "L'assomoir" has bartered his birthright for a mess of pottage, and degraded his undoubted genius by over-copious and hasty writing. It is satisfactory to know that M. Zola "can answer the most foolish question"; he probably has need of this convenient faculty, since we are informed that his doors are always hospitably open to the interviewer. For the sake of parlourmaids in general, it is to be hoped that Mr. Grant Allen's story, "A Self-Respecting Servant," is not founded on fact; but it reads like something that might really have happened. The gem of the illustrations is a reproduction of one of Mr. Sainton's charming silver points—three dainty girl-figures—entitled "Dolce far Niente."

MACMILLAN'S MAGAZINE for this month contains an anonymous article, entitled "Lords and Commons," a temperately-worded plea for the retention of the House of Peers. There is an absence of partizanship about it, and a grasp of essential facts which are not always apparent in the political article. The author may or may not be right in his judgment that the House of Lords possesses all the best requisites of an upper chamber, but his reasons for thinking so are certainly cogent, and are clearly and judicially summed up. Mr. Blackmore's story, "Perlycross," is still continuing; and among the other contents are a short story, entitled "An Oxford Idyll," and a paper on the life of St. Francis of Assisi, the founder of the order of Franciscan monks or begging friars.

THE MAGAZINE OF ART has an interesting contribution by Helene L. Postlethwaite on "Some Rising Artists." Among those of the younger generation of painters who are mentioned are Mr. Frank Brangwyn, who gained that knowledge of the sea, which he has since utilised in his pictures, as a sailor on a coasting vessel; Mrs. Stanhope Forbes (Miss Elizabeth Armstrong); Mr. Charles Sainton, whose silver points have attracted so much notice; Mr. Solomon J. Solomon, the portrait painter; and Mr. Arthur Hacker. There is also an article devoted to the Old Masters' Exhibition at Burlington House, and the customary notes on Art. The frontispiece is an etching from Mr. Waterhouse's picture, "La Belle Dame Sans Merci," which was exhibited in the Academy last year.

### A Sad Occurrence.

THE suicide of a member of the nursing staff at the London Hospital, the first case of the kind which has ever taken place amongst the nurses, occurred last week. Nurse Bevan was fifty-four years of age, a sober, steady, respectable woman, and the jury at the inquest seemed particularly impressed by all the care and attention paid to the afflicted woman, the foreman speaking with kindly appreciation of the consideration which had been shown to her throughout. She had worked at the London Hospital for nearly sixteen years. She was out-patient nurse, and since the beginning of December her condition had caused grave anxiety to those responsible for her. She fancied herself an object of suspicion and contempt, and she could not be persuaded to take a holiday, though this was urgently pressed upon her. The physician, seeing her unwillingness, was of opinion that perhaps if she were permitted to remain at her work she might more easily conquer her obvious depression. However, Nurse Bevan's delusions and excitement appear to have increased, and the matter was laid before the committee on the 29th ult. In spite of all the arguments advanced the poor woman continued to refuse to take a holiday, but on the afternoon of 31st ult. Dr. Frederick Smith decided that she had better take one immediately, although her condition does not appear to have been considered dangerous. A member of the nursing staff who knew her well kindly went to see her widowed sister, and explained that when the nurse went to stay with her next day she must try and cheer her up in every way. It then transpired that for three or four months past Nurse Bevan had confided her delusions to her, and the sister was visibly relieved to find they had no foundation, and that on the contrary the greatest respect and confidence had always been placed in the nurse. On the evening of the 31st ult. Nurse Bevan was quite cheerful at supper time, and it was hoped that the visit to her sister, to commence next day, would greatly benefit her. As she failed to appear at breakfast, however, the night sister at once went to her room, and when the door was forced open she was found unconscious and fully dressed lying on her bed, and although she lived until two a.m. of the next day, she never recovered from the effects of the chloroform which she had taken.

### Where to Go.

PARKES MUSEUM, MARGARET STREET.—Sessional meeting for communications and discussions on sanitary subjects, February 14th, at eight p.m.

DR. C. G. SHELLEY will lecture on "The Feeding of School Boys and School Girls" at four p.m. on February 14th, at 53, Berners Street, in the committee-room of the National Health Society.

SIX lectures on "Nursing" will be given by Sister Katharine at 1, Orme Square, Hyde Park, to commence February 8th, at half-past eleven a.m. Tickets to be obtained at National Health Society, 53, Berners Street.

ST. GEORGE'S HOSPITAL.—A course of lectures to nurses will be given on Tuesdays and Fridays at three p.m. in the board-room of this hospital, commencing on February 16th. Fourteen will be given by Mr. Dent, F.R.C.S.; 6 by Dr. Isambard Owen; and 3 by Dr. Dickinson. Particulars as to fees, &c., can be obtained from the Secretary.

ST. ANNE'S CHURCH, SOHO.—Bach's Passion music will be sung with full orchestral accompaniment at the Friday evening services during Lent at eight p.m. Admittance gratis by tickets, which can be obtained from the Rector, 28, Soho Square, to whom a stamped and directed envelope should be sent.

THE MEDICO-PSYCHOLOGICAL ASSOCIATION will hold its next meeting at the Randolph Hotel, Oxford, on February 15th at ten a.m. At four p.m. Dr. Weatherly will read a paper on "Our Trials and Troubles." Dr. Menzies will open a discussion on "Future Supply and Status of Asylum Nurses and Attendants," and Dr. Chapman will read a paper on "The Causation and Increase of Insanity."

### For Reading to the Sick.

LESSONS OF SICKNESS.

**Motto.**

WHAT smarts, teaches.
Sorrow is surgery.—*Thomas Lynch.*

**Verses.**

Amid my list of blessings infinite
Stands this, the foremost, "That my heart has bled
'Tis Heaven's last effort of goodwill to man;
When pain can't bless, Heaven quits us in despair;
Who fails to grieve, when just occasion calls,
Or grieves too much, deserves not to be blest.
—*Edward Young.*

A little while,
And all things shall be new; the night of earth
Shall pass away for ever; "no more sea"
Shall then be found; for pain and loss and grief
Are swallowed up in radiant victory.
Yet, in the country of eternal spring,
Many shall bend to kiss the Master's feet,
Saying, "He never smiled so sweet before,
Save on the sea of sorrow, when the night
Was saddest on our hearts . . . ."
And only Heaven is better than to walk
With Christ at midnight over moonless sea.—*E. B.*

**Reading.**

Our Heavenly Father is evermore teaching His children not alone through His Word, His Church and Sacraments, but by outward circumstances and inward experiences, by days of brightness and seasons of sorrow, bringing out from each, in turn, manifold lessons for such as with lowly, watchful hearts are ever "waiting upon God."

But if this be so, surely He has special teaching for those on whom He, in His infinite wisdom, has seen fit to lay the cross of sorrow, sickness, or suffering. Those who have watched by the bedsides of the sick, as well as those who have had personal experience of pain and weakness, can abundantly testify to the merciful dealings of our compassionate Father at such times. His lessons to His afflicted children are varied, as are the different characters of those whom, by His loving discipline, He is chastening here, that He may fit them for those blessed mansions above, which He meanwhile is preparing for them.—*M. B. B., from "Voices of Comfort."*

Grief divided is made lighter.—*Seneca.*

There is one lesson which it would be well for all to consider. We read in the vision of the beloved disciple of a "great multitude which no man can number," and that these all came "out of great tribulation." Then, surely, in every generation, through all time, there must be scattered up and down, "of every nation and kindred and tongue" many suffering servants of God. As "heart answereth to heart," so the trial felt by one is oftentimes shared by many, severed and yet united. Let each sufferer, then, think often of the pains and griefs of others, and give them the truest sympathy; and, above all, pray continually for them. We are taught to do this day by day in our Church services, but how few really plead earnestly for others? What a happy link there might be if, while imploring for some alleviation of pain, some lightening of the dark cloud which overhangs themselves, each afflicted one were to ask for a like boon for his fellow sufferers! This, indeed, would be a "golden chain" binding many hearts together.—*M. B. B., from "Voices of Comfort."*

